# Large landslides cluster at the margin of a deglaciated mountain belt

**DOI:** 10.1038/s41598-022-09357-9

**Published:** 2022-04-05

**Authors:** Tomáš Pánek, Michal Břežný, Stephan Harrison, Elisabeth Schönfeldt, Diego Winocur

**Affiliations:** 1 Department of Physical Geography and Geoecology, University of Ostrava, Chittussiho 10, Ostrava, Czech Republic; 2 College of Life and Environmental Sciences, University of Exeter, Penryn, Cornwall TR10 9FE UK; 3 Institute of Geosciences, University of Potsdam, Karl-Liebknecht-Straße 24-25, 14476 Potsdam, Germany; 4 Departamento de Ciencias Geológicas, Facultad de Ciencias Exactas y Naturales, Universidad de Buenos Aires, Intendente Güiraldes 2416, CABA, CP 1428EGA Buenos Aires, Argentina; 5 Instituto de Estudios Andinos (IDEAN), UBA-CONICET, Buenos Aires, Argentina

**Keywords:** Natural hazards, Solid Earth sciences

## Abstract

Landslides in deglaciated and deglaciating mountains represent a major hazard, but their distribution at the spatial scale of entire mountain belts has rarely been studied. Traditional models of landslide distribution assume that landslides are concentrated in the steepest, wettest, and most tectonically active parts of the orogens, where glaciers reached their greatest thickness. However, based on mapping large landslides (> 0.9 km^2^) over an unprecedentedly large area of Southern Patagonia (~ 305,000 km^2^), we show that the distribution of landslides can have the opposite trend. We show that the largest landslides within the limits of the former Patagonian Ice Sheet (PIS) cluster along its eastern margins occupying lower, tectonically less active, and arid part of the Patagonian Andes. In contrast to the heavily glaciated, highest elevations of the mountain range, the peripheral regions have been glaciated only episodically, leaving a larger volume of unstable sedimentary and volcanic rocks that are subject to ongoing slope instability.

## Introduction

Large landslides play major roles in landscape evolution over Quaternary timescales and represent a widespread hazard in high mountains^1^. As mountain glaciers retreat and permafrost thaws, valley slopes can decrease in stability and fail^2^. The physical impact of paraglacial landslides^3^ can lead to a cascade of secondary hazards, such as glacier lake outburst floods^4^ or tsunamis^5^ or any combination of these^6^. In this way, landslides in deglaciating and deglaciated landscapes may pose a threat tens of kilometres downstream from the source area^7^. Glacier recession in the last few decades has led to numerous catastrophic landslides around the world^1,8,9^, some of which have caused hundreds of fatalities^7^. Landslide frequency is predicted to increase locally around mountain glaciers^9^, as well as over larger areas due to the predicted continued recession of larger ice sheets^10^. As a result, to mitigate the risks associated with a predicted increase of mass movements, we need to understand how the spatial distribution of landslides is controlled locally (in alpine valleys) and over regional (mountain belt) scales.

Numerous studies exist on the spatial distribution of paraglacial landslides^11–16^, however very few have evaluated the distribution and controls on landslides at the scale of entire mountain belts or ice sheets^17,18^. Consequently, the complex reasons for slope instability in these deglaciated areas remain somewhat unclear. The distribution of large bedrock landslides in deglaciated areas is assumed to be controlled mainly by the former thickness of ice and the magnitude of glacial decompression^11,17^, post-glacial uplift associated with enhanced seismic activity^14,19^ as well as the distribution of weak rock^12^ and topographic and climatic conditions^13^. As a result, many paraglacial landslides occupy the steepest and most humid portions of deglaciated mountain belts^20^, with a tendency to cluster along seismically active faults^21^.

**Figure 1:**
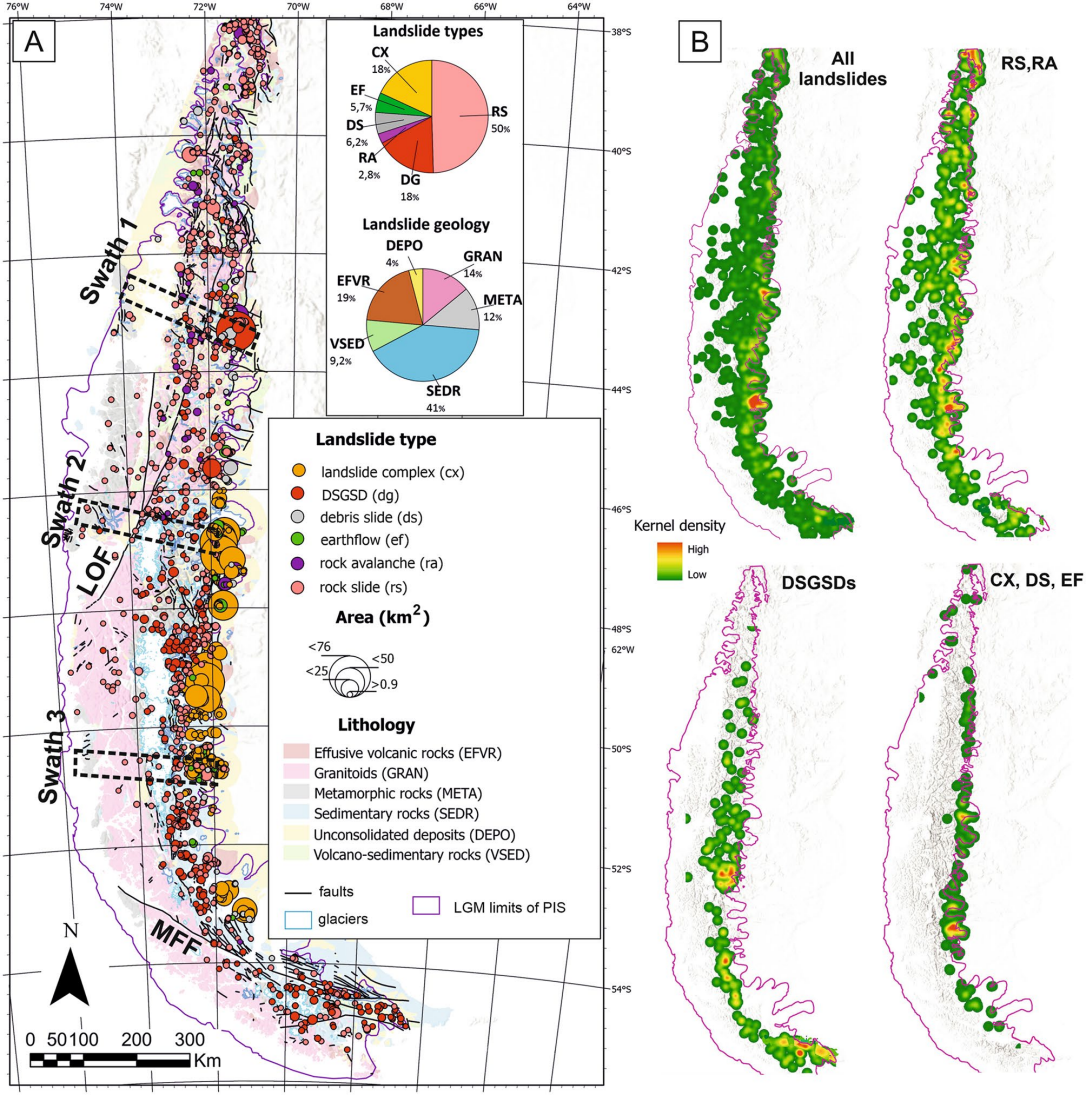
Spatial distribution of large (> 0.9 km^2^) landslides and geology within the LGM limits of the PIS. (**A**) Landslides area displayed as size-graded centroids with inset pie diagrams showing the proportion of landslide types and their lithology. Geology is from the 1:1,000,000 maps of Chile (SERNAGEOMIN, 2000) and Argentina (SEGEMAR, 1995). (**B**) Kernel density maps for all landslides and their individual types. Major faults: LOF—Liquiñe-Ofqui fault zone, MFF—Magallanes-Fagnano fault.

Here we focus on the spatial distribution of large (> 0.9 km^2^) landslides in the deglaciated portions of Patagonia (~ 305,000 km^2^)^22^ and show a radically different pattern. Our mapped area stretches for ~ 2000 km along the southernmost portion of the Andes (Patagonian and Fuegian Andes) between ~ 38°S and 56°S (Fig. 1) and includes the area covered by the Last Glacial Maximum (LGM) Patagonian Ice Sheet (PIS). We demonstrate that the largest landslides in Southern Patagonia preferentially occupy lower, tectonically less active, and arid parts of mountain belts. We argue that this arises since the peripheral parts of the mountain ranges have not been as heavily glaciated as their central massifs, and potentially unstable rocks have not been effectively removed by glacial activity during the Quaternary.

## Regional settings

Our mapped area stretches for ~ 2000 km along the southernmost portion of the Andes (Patagonian and Fuegian Andes) between ~ 38°S and 56°S (Fig. 1). It is outlined by the local LGM limits of PIS as reconstructed by Davies et al.^22^ and comprises three distinct domains (from west to east): a dissected fjord landscape with archipelagos (e.g., Chiloé and Tierra del Fuego); the spine of the Andes with the highest elevations ranging between 3000 and 4000 m a.s.l., and the eastern flatter piedmont zone. The area hosts some of the largest contiguous extrapolar ice fields, such as the Northern Patagonian Ice Field (NPI; 3976 km^2^) and the Southern Patagonian Ice Field (SPI; 13,219 km^2^), and numerous smaller ice caps and mountain glaciers (Fig. 1)^23^. The regional climate is influenced by the Southern Westerly Winds (SWW) bringing abundant precipitation from the Pacific Ocean to the western flank of the orogen (> 5000 mm/yr), while the eastern piedmont is in the rain shadow and receives < 500 mm of precipitation annually^24^.

The geological evolution of the area has been driven by the interplay between the Nazca, Antarctic, South American and Scotia plates (Fig. 1). The Nazca plate north of the Chile Triple Junction has been subducted in a northeast direction beneath the South American plate at 66 mm/yr, whereas the southern Antarctic plate underplates eastward at about 20 mm/yr^25^. The geology of the PIS region comprises three major zones: (1) Basement made of Paleozoic metamorphic rocks and calc-alkaline Jurassic-Neogene granitoids (Patagonian Batholith) forming the western coast and axial chain of the Andes^26^; (2) Mesozoic and Cenozoic sedimentary and volcanic rocks building a retroarc wedge in the eastern Patagonian Andes and most of the Fuegian Andes^27^, and (3) sedimentary rocks and Plio-Pleistocene back-arc flood basalts^28^, forming tablelands with flat-topped mesetas along the eastern piedmont of Andes (Fig. 1). Holocene volcanic activity follows mainly the axial chain of the Andes; some of the most active volcanoes such as Villarica (2847 m a.s.l), Calbuco (2015 m a.s.l) and Chaitén (1122 m a.s.l) have experienced major eruptions in the last two decades^29^. Most of the seismic activity is distributed offshore and in the northeastern part of the region (Fig. 1), which was affected by the 1960 Mw 9.5 Valdivia megathrust earthquake. The northern half of the Patagonian Andes is dominated by the fast-slipping (~ 11.6–24.6 mm/yr) dextral Liquiñe-Ofqui fault^30^; the source of the 2007 Mw 6.2 Aysén Fjord earthquake^31^. The major tectonic structure in the southern region is the Magellanes-Fagnano fault system representing a sinistral boundary between the South American and Scotia Plates, with estimated movement rates ~ 7.8–10.5 mm/yr^32^.

Patagonia has experienced repeated glaciations for over ~ 6 Ma^33^, leaving a landscape of deeply incised valleys, fjords, cirques, and mountain ridges in the Andes, and some of the world´s largest terminal moraines and outwash plains in the piedmont zone^22,34^. During the LGM, locally dated to ~ 35 ka^22^, the PIS, covered about 480,000 km^2^. Numerous fast-flowing ice lobes drained the PIS both to the west and east. The PIS began to recede from the foothills to the Andes ~ 18 ka ago and left numerous glacial lakes at its front^22,35^. By ~ 15 ka, the PIS had been largely separated to form individual ice fields^22^, causing repeated catastrophic drainages of glacial lakes to the Pacific Ocean^35^. Due to glacial-isostatic adjustment in response to glacier recession, the PIS region has recently experienced some of the world’s fastest vertical crustal movements, peaking at 41 mm/yr in the northern part of the SPI^36^.

## Methods

### Landslide mapping

We mapped large landslides over an area of ~ 305,000 km^2^, representing the land area (excluding modern glaciers and lakes) within the LGM boundaries of the PIS^22^. We arbitrarily considered large landslides as those with a total area A_L_ ≥ 1 km^2^; however, due to uncertainty in landslide delimitation and in order not to omit landslides approaching 1 km^2^, we lowered landslide area limit to 0.9 km^2^. We utilized ESRI™ World Imagery Layer providing satellite images from DigitalGlobe (Maxar), and shaded relief based on the WorldDEM4Ortho with pixel size 24 m. DigitalGlobe (Maxar) covers the PIS area with a mosaic of images from QuickBird-2, GeoEye-1, and WorldView2-4 satellites with a resolution of 0.3–0.6 m captured between 2004 and 2021. For better visualization and mapping of the landslides through the oblique perspective, we also used Google Earth Pro imagery. Landslides were mapped using common criteria for identification (e.g., presence of arcuate scarps, tension cracks, closed depressions, bulges and lobate toes^37^), and they were classified as rock slides (both planar and rotational), deep-seated debris slides, earthflows, rock avalanches, and deep-seated gravitational slope deformations (DSGSDs; Supplementary Fig. 1). Coalesced and superimposed landslides were mapped separately; although this criterion was difficult to meet in the eastern part of the area, where rock slides and spreads are overlapped by multiply generations of earthflows forming continuous rims along the volcanic mesetas^38^. Therefore, such features are classified as landslide complexes. For visualization purposes, the spatial distribution of landslides is displayed with kernel density maps calculated from landslide centroids with a 20 km circular window^39^. Landslide metrics were extracted in ArcGIS Pro and the landslide area was used in the analyses of landslides distribution. For each landslide, the maximum age was determined by the deglaciation of its site according to Davies et al.^22^.

### Searching for landslide controls

To identify the influence of possible landslide controls, we analyzed geological and tectonic conditions (involving seismicity, recent uplift and long-term erosion), topography and distribution of precipitation (Supplementary Fig. 2). From topographic characteristics we used local relief, slope, hypsometric integral and residual relief, which were calculated from NASADEM global digital elevation data at a nominal resolution of ∼ 30 m (https://lpdaac.usgs.gov/products/nasadem_hgtv001/). Local relief was calculated within a 5 km circular window to capture the common wavelength of the topography. Residual relief is defined here as the difference between the DEM surface and a base-level surface interpolated with the Inverse Distance Weighting (IDW) algorithm from the elevation of the channel network with an upslope contributing area larger than 2.5 km^2^. Geology was digitized from the 1:1,000,000 maps of Chile (SERNAGEOMIN, 2000) and Argentina (SEGEMAR, 1995) and the fault pattern was complemented by newly identified Quaternary faults from Georgieva et al.^40^. Lithologies mapped in geological maps were simplified into six different rock units (Fig. 1). Potential exposure of individual landslides to regional seismicity was approximated by the calculation of Arias Intensity^41^. We followed the approach of Crosta et al.^17^ and calculated sum of Arias Intensity of sufficiently strong earthquakes (based on M_s_ and distance^42^) for given 50-km^2^. We obtained earthquakes (Ms ≥ 3) from the USGS Earthquake Catalog (https://earthquake.usgs.gov/earthquakes/search/). To determine the position of landslides relative to the long-term erosion of the area, Apatite Fission Track (AFT) ages were interpolated from Thompson et al.^43^, Rojas Vera et al.^44^, and Goddard and Fosdick^45^. We also considered published AHe ages^27,40,43,45^, but did not interpolate them due to the uneven coverage of the area. Recent uplift rates for the central part of the PIS area (surroundings the NPI and SPI) were extracted from Richter et al.^36^. We characterize first-order climatic patterns of the area as annual precipitation totals estimated for the period 1970–2000^46^ (available from WorldClim.org).

The influence of individual environmental variables on landslide distribution was investigated by Principal Component Analysis (PCA). For this purpose, the PIS area was divided into a 50-km^2^ grid clipped by PIS limits and coastlines, where the dependent variable is the percentage of landslide coverage, and the independent variables are topographic, geological, and climatic characteristics (Supplementary Fig. 2). All topographic data, along with annual rainfall, AFT age, and fault density, were used as average values, while lithology was expressed as % cover of a given rock type within the clipped 50-km^2^. As the study area of the PIS has a very irregular boundary determined by the rugged fjord coastline and the bay-like arrangement of the LGM limits, some of the polygons resulting from the clipped 50-km^2^ occupy only a very small area (Supplementary Fig. 2). Therefore, only polygons with an area greater than 10% of the original 50 km^2^ were included in the statistical analysis (i.e., total 210 squares > 250 km^2^ each).

## Results

### Spatial distribution of landslides

We mapped 1457 large landslides within the LGM limits of the PIS, and large landslides associated with ice-contact surfaces at the margins of the LGM ice sheet (Fig. 1). The size of individual landslides range between 0.9 and 71 km^2^ (Fig. 1) and 10% of the largest landslides amount for nearly half (44%) of the total landslide area. Landslide distribution is spatially clustered, with most landslides affecting the eastern piedmont of the Patagonian Andes (Fig. 1). One third of the mapped landslides (both by number and area) cluster within 10 km of the eastern LGM margin, with peak landslide densities located around 43°, 47° and 51°S (Fig. 1). Clusters around Lago Buenos Aires and Lago Argentino (47°–51°S) cover < 3% of the PIS area but comprise 22% of the landslide population (28% by area). Large landslides are almost absent in the western Patagonian Andes and in the fjords (Fig. 1).

Landslide types are dominated by rock slides (50%), landslide complexes (18%) and deep-seated gravitational slope deformations (DSGSDs; 18%), followed by debris slides (6.2%) and earthflows (5.8%; Fig. 1; Supplementary Fig. 1). There is a scarcity of long-runout landslides with only 41 rock avalanches (2.8%) and only 34 landslides (2.3%) dammed valley floors. Type-specific landslide densities mostly follow the overall pattern of landslide distribution, although DSGSDs cluster NE of the SPI and in the Fuegian Andes (Fig. 1).

Most landslides originated in volcanic and sedimentary rocks, involving > 70% of the total landslide population (Fig. 1; Supplementary Table 1). Effusive volcanic rocks forming plateaus along the eastern piedmont of Andes are most affected, with landslides forming ~ 8% of their area. In contrast, landslides within the Patagonian Batholith (granite), building the highest elevation of the Andes, cover an area of less than 0.5%. Only ~ 8% of large landslides are within 1-km distance from mapped faults and approximately half of them lie more than 10 km from faults (Supplementary Fig. 3). One cluster of landslides, mainly DSGSDs, is situated along the Magallanes–Fagnano fault in the Fuegian Andes (Fig. 1). This is the only case where the occurrence of large landslides in Patagonia overlaps with a major active fault. Large landslides are nearly absent along the Liquiñe-Ofqui Fault, recently recognized as one of the world’s fastest moving strike-slip faults^30^.

**Figure 2:**
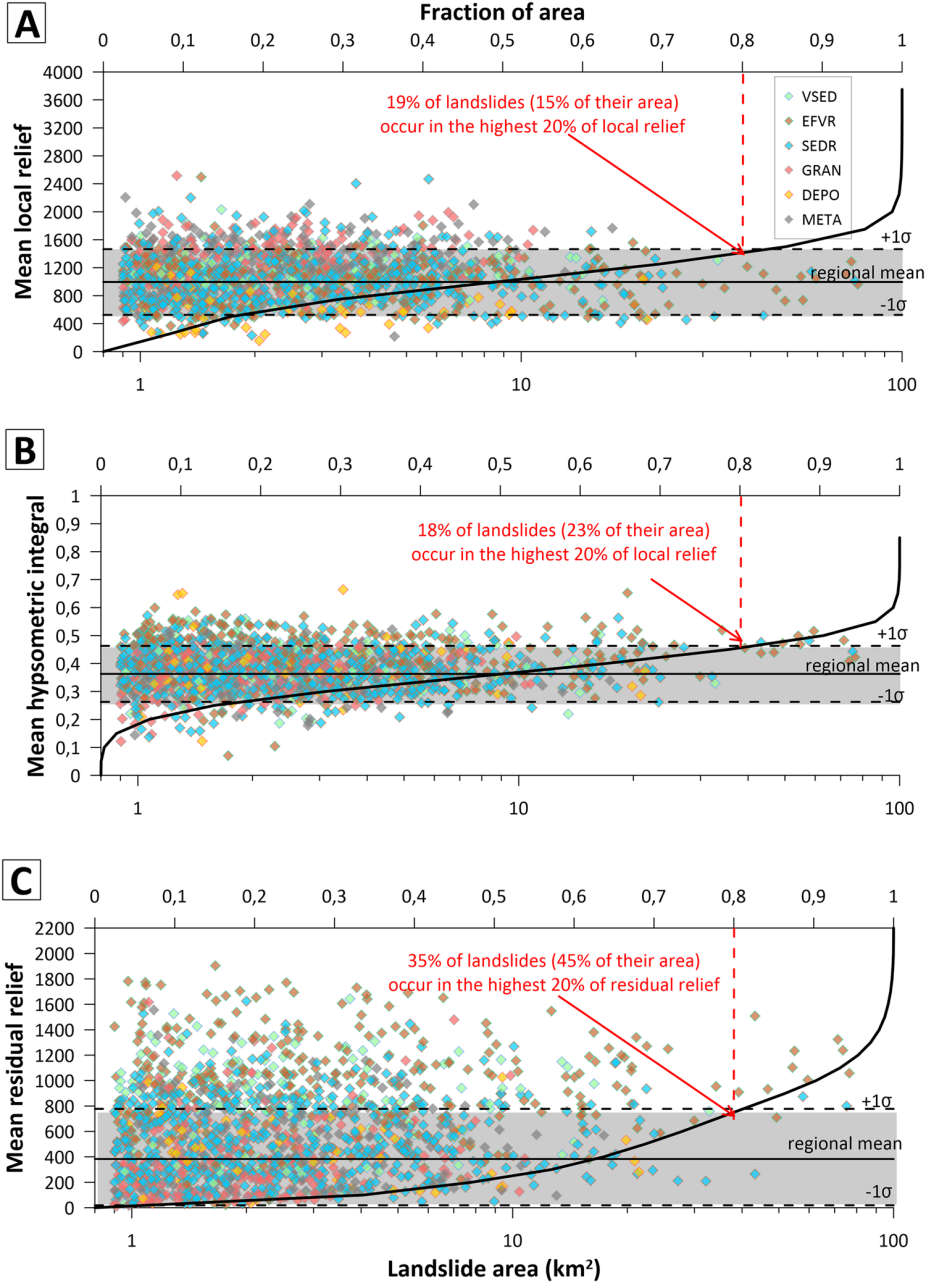
Relationship between large landslide areas and (**A**) local relief, (**B**) hypsometric integral and (**C**) residual relief calculated as mean values within 5-km buffers around landslide centroids. Landslides are stratified according to their dominant lithology (see Fig. 1A for an explanation of the abbreviations.). Black curves are cumulative distributions of local relief, hypsometric integral and residual relief respectively within the PIS region.

The influence of topographic parameters on the distribution of landslides is less clear. Although there is no correlation between landslide area and these topographic characteristics, more large landslides occur in regions characterized by high residual relief (Fig. 2). About 35% of the total population and 45% of total landslide area is concentrated within the highest 20% of residual relief, but in the case of local relief and hypsometric integral, most landslides are within 1σ of their regional means (Fig. 2).

**Figure 3:**
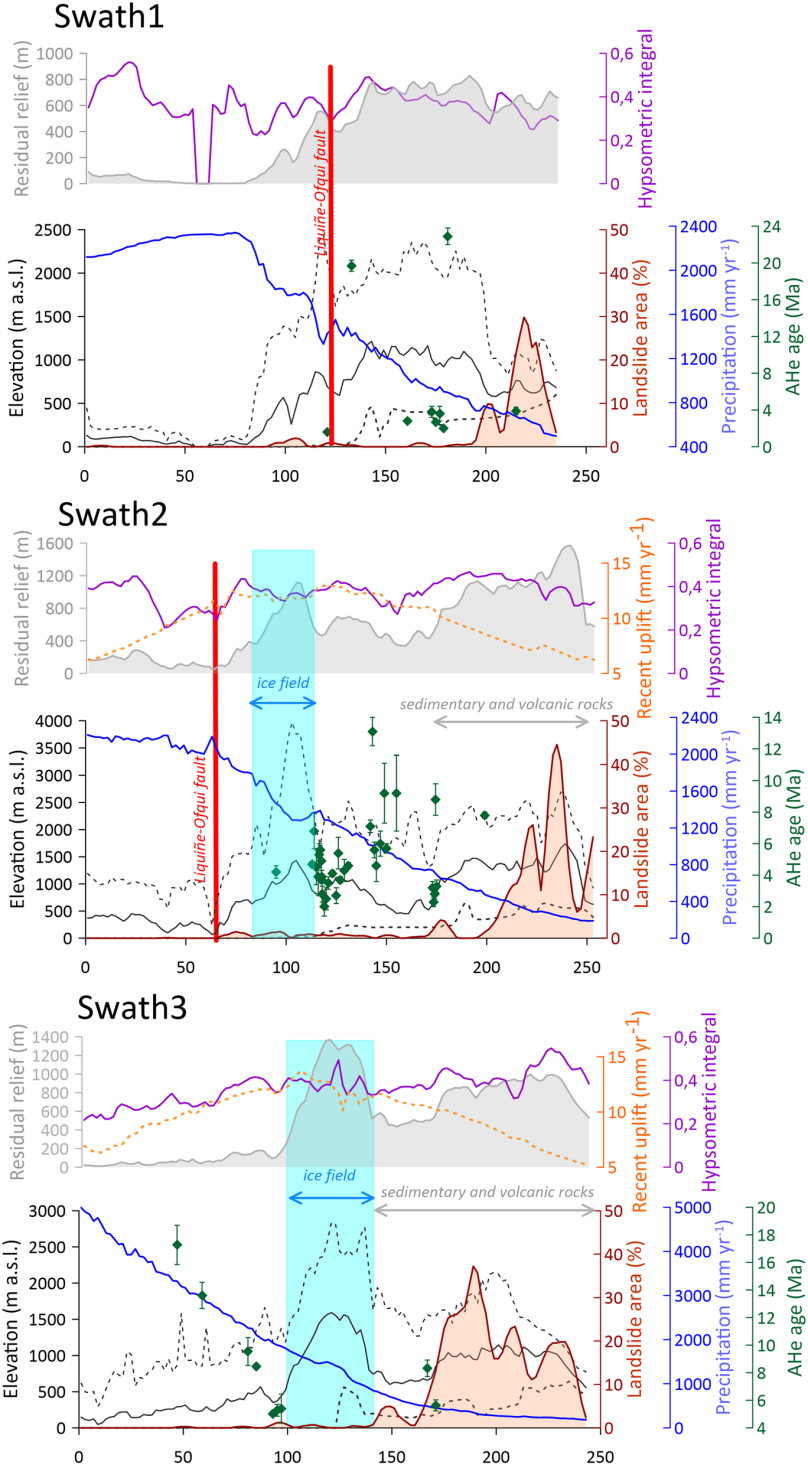
Three 50-km wide swath profiles across the PSI region. Selected landscape variables are plotted against the percentage of area covered by large landslides. Elevation, hypsometric integral and residual relief are calculated from NASADEM, mean annual precipitation totals are from WorldClim.org^46^ and recent uplift is according to Richter et al.^36^. AHe ages are from Thompson et al.^43^, Fosdick et al.^27^, Georgieva et al.^40^ and Goddard and Fosdick^45^. For locations of swath profiles, see Fig. 1A.

Three swath profiles constructed across most prominent landslide clusters (see Fig. 1 for location) show that regions with the highest landslide density closely coincide with the highest residual relief. However, not all domains with high residual relief host large landslides (Fig. 3). In contrast, the distribution of landslides is less dependent on elevation, local relief and hypsometric integral and there appears to be no relation with the long-term erosion rates documented by highly scattered AHe ages. Recent uplift rates increase towards the centre of the mountains and the modern ice fields^36^, i.e., opposing to landslide density (Fig. 3; Supplementary Fig. 4). An inverse relationship also exists between landslide occurrence and precipitation totals, with the highest density of large landslides in the arid eastern periphery of the Patagonian Andes (Fig. 3).

### Timing of landslides

**Figure 4:**
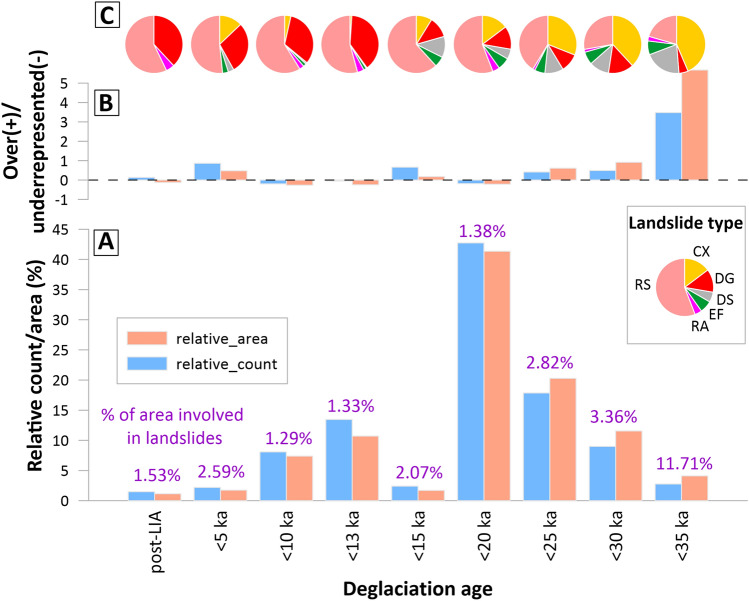
Distribution of large landslides in regions with different ages of deglaciation. (**A**) Bar plot of the large landslide relative area/count in the deglaciation period. Number above histograms illustrates percentage of the area which is affected by large landslides. (**B**) Quantification of landslide over/under-representation. Negative values suggest an under-representation of the landslides while positive values suggest an over-representation of landslides in landscape strips with different ages of deglaciation. (**C**) Proportion of landslide types in regions with different ages of deglaciation. The presence of landslides in distinct deglaciated areas approximates their maximum age. Note the considerable increase in large landslide area and number in landscape strips which were deglaciated ≥ 20 ka, coinciding with an increase in the proportion of volcanic and weak sedimentary rocks. The ages of deglaciation are after Davies et al.^22^.

After assuming that the PIS erased all traces of older landslide deposits, the location of landslides and the timing of deglaciation (according to Davies et al.^22^) indicates the possible maximum landslide age. We do not observe an increase in the number of landslides with the length of time elapsed since ice retreat (Fig. 4). Most of the landslides (more than 40% by number and area) are located in the area where deglaciation occurred between 20 and 15 ka, but this area occupies more than 50% of the PIS area, so landslides are slightly under-represented here. Considering the contribution of surface area of individual deglaciation zones, landslides are over-represented especially in areas where ice retreat took place before 35–30 ka and 5–0.2 ka; the former overlaps exclusively with weak volcanic and volcano-sedimentary rocks. The percentage of area involved in landslides (~ 1–3%) is similar to the majority of deglaciation intervals, with the exception of the area covered by the oldest period of deglaciation (35–30 ka) in our inventory, where landslides represent almost 12% of the area (Fig. 4).

In older deglaciated landscapes, there is a higher proportion of landslide complexes and earthflows, while areas deglaciated during the Holocene are more prone to DSGSDs and rockslides. This may reflect the geology and topography of particular deglaciated land strips (e.g., high susceptibility to earthflows by effusive and sedimentary rocks which were deglaciated before the Holocene^38^), but also the time required for the evolution of landslides. The dominance of short traveled rock slides and DSGSDs in later deglaciated areas may indicate insufficient time to develop catastrophic landslides through progressive failure; a process that can last up to ~ 10 ka in mountain areas^47^.

### Multivariate analysis of landslide controls

We divided the PIS area into 50-km^2^ for which landslide coverage (%) and 14 independent landscape variables were calculated (Supplementary Fig. 2). The percentage of large landslide areas in individual squares shows a positive correlation with residual relief (Spearman's rank correlation coefficient *r*_*s*_ = 0.672). Landslide coverage also correlates with the percentage of area occupied by sedimentary (*r*_*s*_ = 0.493) and volcanic-sedimentary rocks (*r*_*s*_ = 0.451), and reveals a negative correlation with annual precipitation totals (*r*_*s*_ = − 0.547; Supplementary Fig. 5).

**Figure 5:**
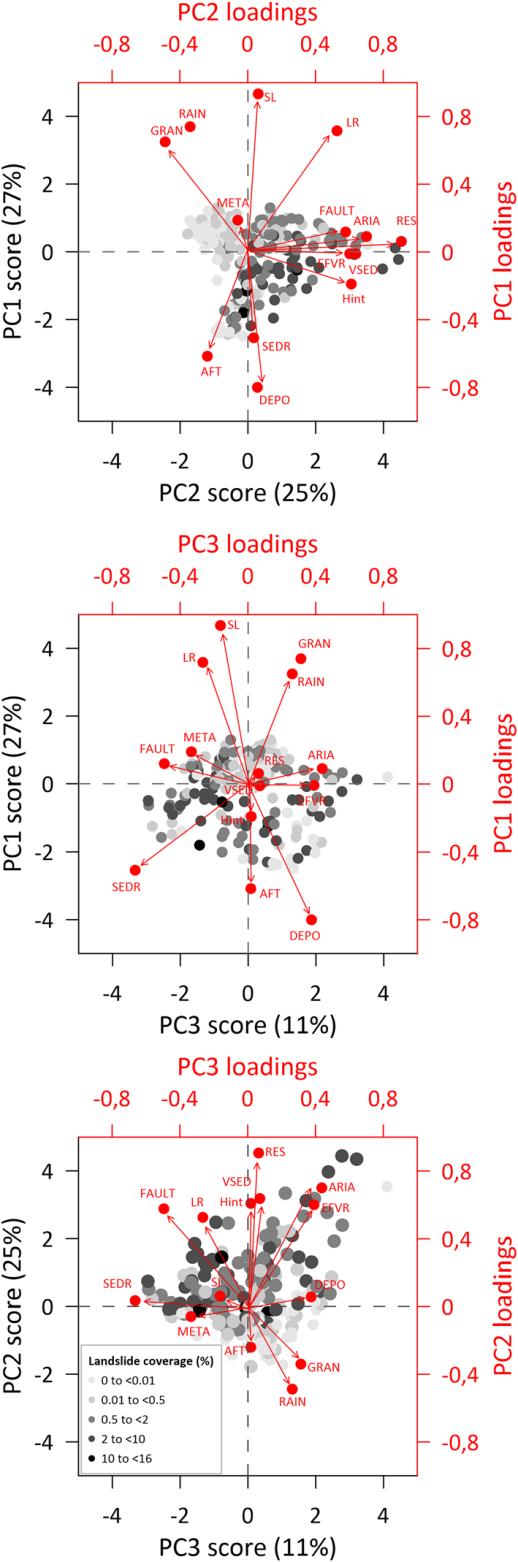
PCA results showing principal component scores and loadings for the first three principal components (PC1–PC3). Labels abbreviations: ARIA: Aria Intensity, AFT: mean AFT age, DEPO: coverage (%) by Quaternary unconsolidated deposits, EFVR: coverage (%) by effusive volcanic rocks, FAULT: mean fault density, GRAN: coverage (%) by granitoid rocks, Hint: mean hypsometric integral, LR: mean local relief, META: coverage (%) by metamorphic rocks, RAIN: mean annual precipitation totals, RES: residual relief, SL: mean slope, SEDR: coverage (%) by sedimentary rocks; VSED: coverage (%) by volcanic-sedimentary rocks.

To establish the relative importance of landscape controlling variables, we performed Principal Component Analysis (PCA) on the data. Our PCA scores are color-coded by landslide coverage in individual squares, which establishes the weight of individual independent variables^17^ (Fig. 5). The first four principal components with eigenvalues higher than 1 account for 71% of the entire multivariate space variance and first three principal components (PC1–PC3) shown in Fig. 5 explain 63% of the multivariate space variance. PC1 is associated with slope (SL), local relief (LR), granite occurrence (GRAN), rainfall (RAIN), presence of unconsolidated Quaternary deposits (DEPO) and AFT age (AFT). From variables with high positive PC1 loadings, GRAN and RAIN are oriented in the direction of large landslide density decreases, suggesting that they negatively contribute to distribution of landslides. Other variables have rather minor (LR, DEPO), or no influence on landslide occurrence (AFT), as they are placed diagonally or perpendicular to the main landslide coverage trends (Fig. 5). PC2 involves mainly variables with high positive loadings positively influencing landslide coverage, such as residual relief (RES), hypsometric integral (Hint), Arias Intensity (ARIA), fault density (FAULT) and occurrence of volcanic (EFVR) and volcanic-sedimentary rocks (VSED; Fig. 5). Clustering of these variables in the direction of landslide coverage increase is demonstrated especially by PC1/PC2 and PC2/PC3 plots (Fig. 5). RAIN within PC2 is oriented in the direction of landslide density decrease. PC3 has an association only with the occurrence of sedimentary rocks (SEDR) favouring distribution of large landslides (Fig. 5). The PCA thus suggests that faulted sedimentary and volcanic rocks, with moderate local relief, and wide-ridge topography within the seismically active and rather dry zone, represent the most landslide prone areas within the PIS region. In contrast, areas of steep relief with high annual rainfall, underlain by strong granitic rocks, are least affected by large landslides.

## Discussion and conclusion

Our study provides the first extensive ice-sheet scale inventory of large landslides; previous research has presented only parts of deglaciated mountain ranges^16^ or focused just on particular landslide types^17^. As landslide morphology cannot survive repeated glaciations, our landslide population within the PIS developed over the last ~ 35 ka^22^. Landslide coverage in areas with different ages of deglaciation does not differ significantly, which may indicate relatively rapid landslide origin after ice retreat. If landslides formed gradually, their share would increase with the time elapsed since deglaciation, which is not the case in our inventory. This scenario would be consistent with the so-called exhaustion paraglacial model of Cruden and Hu^48^, which assumes that deglaciated areas contain a finite number of potential failure sites, the number of which is progressively reduced over time. However, radiometrically dated landslides are few in Patagonia and have occurred both just after deglaciation^49^ and with a lag of many millennia^38^, so an extensive dating campaign of Patagonian landslides will be needed to verify whether the "exhaustion model" is valid in the region.

Although landslide coverage within the LGM limits of PIS (1.9%) is similar to other mountain ranges that have undergone deglaciation over a similar time interval, such as the Southern Alps in New Zealand (2%)^13^, the Pyrenees (1.8%)^50^, the Carpathians (1.1%)^16^, and slightly less than the European Alps (5.6%)^17^, this relatively high value is due to a small number of landslide hotspots located outside of the Patagonian Andes along the eastern edge of the PIS. Most of the mountains in the PIS area have a small fraction of landslides (< < 1%), resembling tectonically less mobile Paleozoic orogenic belts (e.g., British Mountains with 0.8% landslide coverage^18^). The PIS inventory differs from other young mountain belts also in the absence of catastrophic rock avalanches. Although some individual rock avalanches have been described in the area^51^, long-runout catastrophic landslides associated with landslide dams are much more abundant in other Cenozoic orogens^20^. The rarity of rock avalanches in the crystalline part of the PIS region is another feature more reminiscent of Palaeozoic orogens such as the British and Scandinavian Mountains^18^.

Thus, the near absence of large landslides in one of the world's most humid, tectonically active and glacio-isostatically mobile mountain belts is surprising. The influence of major fast slipping faults (esp. Liquiñe-Ofqui Fault^30^) on the distribution of large landslides in Patagonia is negligible and large landslides cluster in the semiarid piedmont of mountains characterized by rather low recent uplift rates^36^. This landslide pattern differs in comparison with other reported deglaciated orogens. In the Swiss Alps, for example, the largest concentration of landslides overlaps with areas revealing the highest postglacial uplift^52^. However, in the Swiss Alps, the zone of highest uplift coincides with weak schists and flysch rocks^52^, as opposed to the Patagonian Andes, where it is in a zone of competent granitic batholith^36^. Although the absence of large landslides along major faults is clear (Fig. 1), many may have occurred along minor faults or joint systems that are not marked in geologic maps. Supported by the analysis of the PCA, fault density in 50-km^2^ correlates positively with landslides, suggesting that intensively faulted regions are more prone to large landslides. The role of earthquakes is also somewhat ambiguous. Even though the PCA shows seismic activity as one of the influencing factors of landslide distribution, the absence of large landslides in the western part of the PIS region implies that it mainly acts as a trigger for large landslides outside the granite domain. This is well evident to the north of the study area, where once the northern branch of the Liquiñe-Ofqui Fault system enters the volcanic and sedimentary rocks, it predisposes a cluster of several landslides greater than 1 km^3^ in size^53^. Besides the presence of strong rocks, the reduced effect of earthquakes on landslide genesis in the highest/western part of the Patagonian Andes may also be due to the fact that this area was glaciated the most and for the longest time. Previous studies show that ice may reduce seismic intensity and therefore decrease the adverse effects of earthquake shaking on slope stability^54^.

We did not find any relationship with long-term rock uplift and denudation. In contrast to the European Alps, where DSGSDs occupy mostly landscape domains with average AFT ages^55^, most pronounced clusters of large landslides in the PIS region overlap both with oldest and youngest AFT domains. For example, the landslide gap around 44°S coincides with the so-called Patagonian Erosion Hotspot, characterized by anomalously young thermochronological ages suggesting fast erosion in the last 2 Ma^56^. However, the correlation of landslide occurrence with thermochronological data is problematic because coverage of AFT and AHe ages is spotted (Fig. 3).

**Figure 6:**
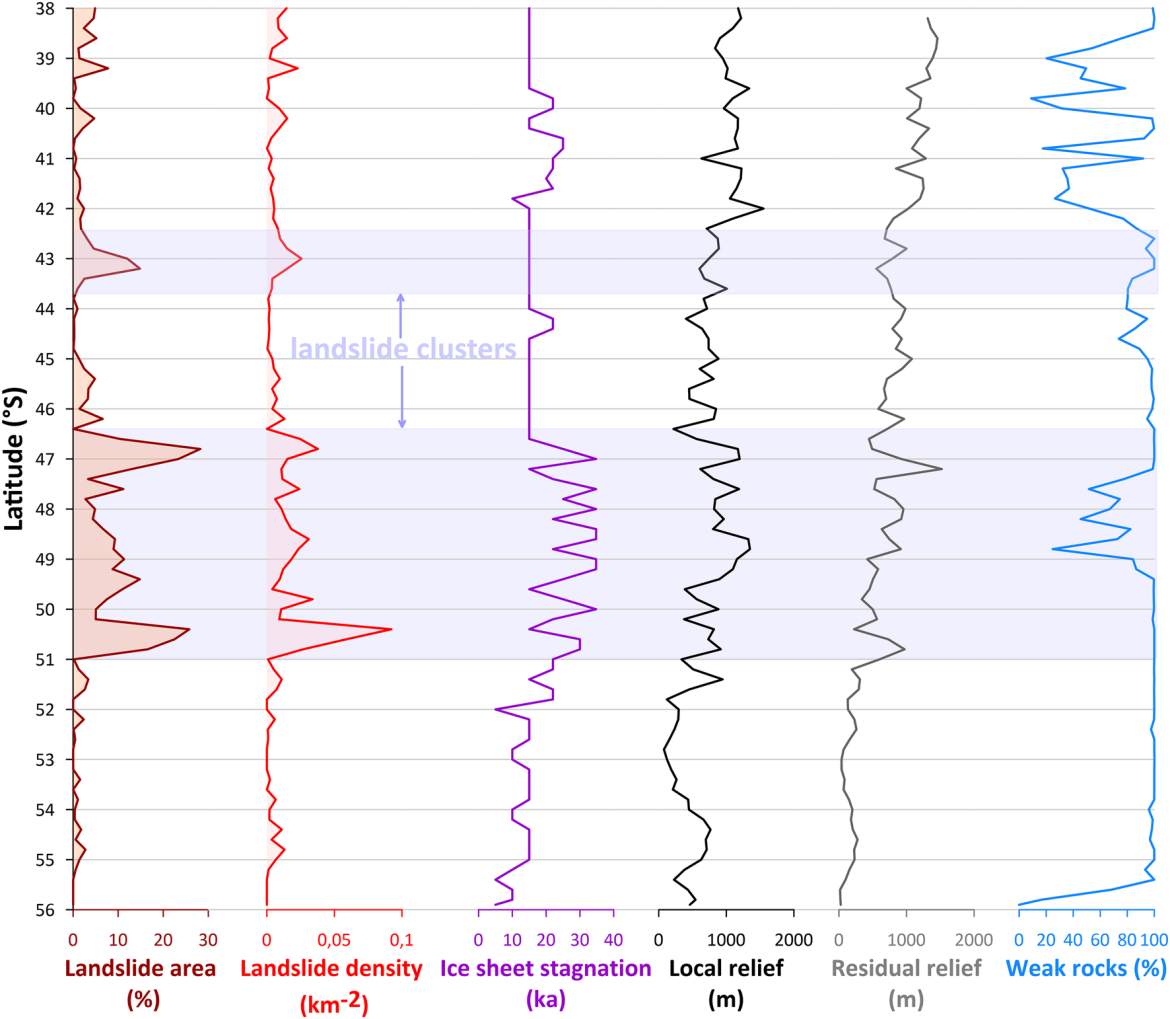
Distribution of large landslides (expressed as landslide area and density) within 10-km-wide zone along the eastern LGM margin of the PIS, plotted against selected landscape variables calculated for 2° bins of latitude. Ice sheet stagnation means how long ice margin stayed within 10-km wide belt from the 35 ka LGM outline (calculated as subtraction of the youngest age of glacier presence within the given bin from 35 ka). Topographic parameters (local relief, residual relief) were calculated from NASADEM. Weak rocks are all except crystalline (plutonic and metamorphic) rocks.

Although large landslides are more abundant along the eastern margin of the Patagonian Andes, their distribution is not uniform (Fig. 6). The southernmost belt (56–51°) with low landslide occurrence corresponds to the lowest values of “ice sheet stagnation” (defined here as the time that the front of the PIS was within 10 km of its maximum limit ~ 35 ka ago), as well as local and residual relief, although weak sedimentary and volcanic rocks cover almost 100% of this domain. The major “landslide belt” between 46° and 51°S overlaps with the highest “ice stagnation” values, high local relief, and major peaks of residual relief. Weak rocks underlie < 50% of the northern part of this domain, suggesting that lithology is not the only landslide factor in this area. Northward from 46°S isolated landslide peaks are largely independent from topography and duration of ice sheet front, but mostly correspond with the distribution of weak rocks (Fig. 6). Therefore, the distribution of large landslides along the eastern margin of Patagonian Andes seems to be controlled mainly by a combination of sufficient local and residual relief with the presence of weak rocks and the vicinity of the ice sheet front. Most (and the largest) landslides occur where there is a relatively high local relief^57^ but also a large volume of potentially unstable rocks^58^, approximated by high residual relief values. Field studies show that the main geological preconditions for large landslides along the eastern margin of the Patagonian Andes are contacts of rigid and incompetent rocks (e.g., Plio-Pleistocene basalts overlying Miocene sediments^38^), whereas in the crystalline part of the PIS area it is mostly schistosity^53^ or brittle fault planes^59^.

The question is to what extent the formation of landslides in the PIS area was related to cryospheric factors. Although we do not have data about the thickness of PIS, the concentration of large landslides along the margin of the PIS indicates that glacier thickness and debuttressing during glacier retreat were not a major factor for genesis of landslides. Permafrost may have contributed to the stability of the slopes^60^, but this has been virtually absent in the Patagonian Andes in recent times^61^, unlike in the higher Central Andes where its degradation affects the formation of rockslides^62^. The influence of permafrost thawing on landslides cannot be ruled out in earlier post-deglaciation periods, but its influence can only be assessed once a high-resolution spatiotemporal model of its evolution is obtained, and the age of landslides is better understood^60^. Nevertheless, the relatively close and prolonged position of the ice front in the landslide clusters could have affected slope stability in many ways, such as repeated buttressing/debuttressing along the same slope sections^63^, seismicity due to glacioisostasy^19^, meltwater action^4^, the development of forebulges and climatic influences^2^ including permafrost degradation^60^. Furthermore, landslide clusters at 43° and between 47°and 51° spatially coincide with the existence of large glacial lakes, which expanded between ~ 18 and 10 ka^22^. The coincidence in time between the existence of glacial lakes and the formation of some of the largest landslides in the PIS around Lago Buenos Aires and Lago Pueyerredón has been reported by Pánek et al.^49^. Cross-cutting relationships of landslides with paleoshorelines suggest that some landslides originated during rapid drawdowns of glacial lakes due to their catastrophic drainages to the Pacific Ocean^35^.

**Figure 7:**
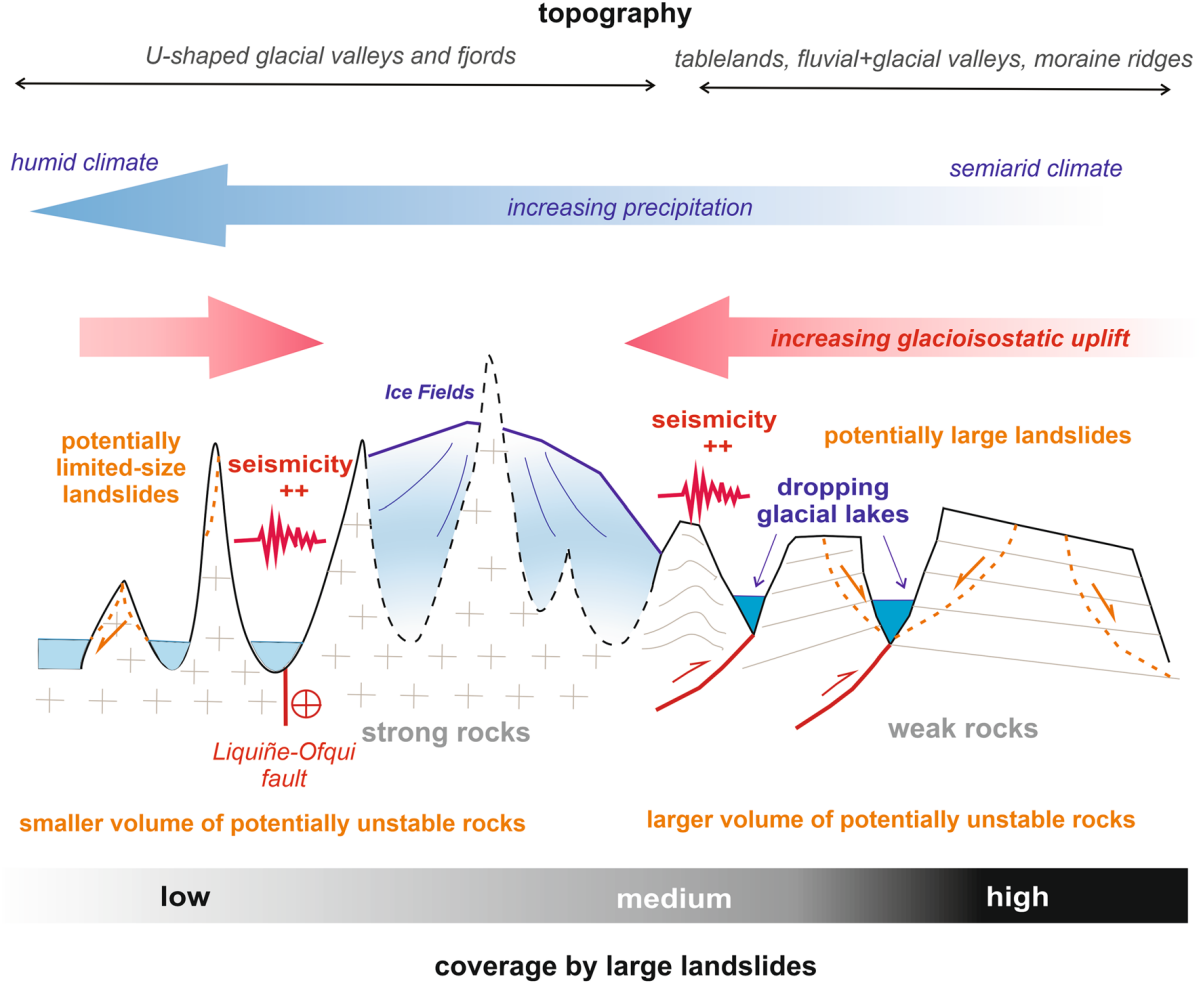
Conceptual scheme showing tendency of large landslide distribution in a typical W-E transect across the PIS region.

The asymmetry in the distribution of landslides in the PIS area and their almost complete absence in the highest alpine part of the mountain range can be explained by the dominance of strong granitic rocks of the Patagonian Batholith and deeply incised glacially sculpted topography along the western fjords and backbone of the Patagonian Andes (Fig. 7). Although this area is exposed to potentially strong triggers such as seismic activity^51^ or high precipitation^64^, the topography here is less prone to large landslides because it has been glacially modified for the last ~ 6 Ma^33^, representing one of the longest records of mountain glaciation in the world. Matured glacial valleys underlain by crystalline rocks are well adapted to efficient ice discharge^18,65^ and are therefore less prone to major slope instabilities than the eastern side of the deglaciated orogen, where glaciations progressed only episodically and left large volumes of potentially unstable sedimentary and volcanic rocks uneroded (Fig. 7). Furthermore, an offshore calving ice front along the west coast of Patagonia did not have such an impact on paraglacial slope stability as the land-terminating eastern margin of PIS. However, this concept is valid only for large bedrock landslides, not for shallow slides and smaller rockfalls, which are numerous in the highest and western part of the Patagonian Andes, especially in the area deglaciated after the LIA^65^ or around active volcanoes^66^. The PIS region can thus be divided into two domains with respect to frequency-magnitude, types and triggers of landslides (Fig. 7). The western, higher part that coincides with the fjord and crystalline rocks region contains mainly smaller shallow slides that are triggered by both earthquakes and heavy rainfalls. These landslides occur here frequently at the present time^51,64^. However, due to the small volume of potentially unstable rocks above the base level and the predominance of strong rocks, large landslides are almost absent here. In contrast, the eastern margin of the PIS region is dominated by large landslides in weak rocks. These landslides are mostly ancient and recent landslide activity is limited here^49^. We assume that these large landslides were triggered mainly due to high-magnitude events in the transient period after deglaciation, e.g. due to seismic activity related to glacial isostatic rebound^49^, rapid drawdowns of glacial lakes^49^, and possibly also due to extreme hydro-meteorological events in the more humid phases of the Late Glacial and Holocene^38^.

We conclude that the distribution of landslides over large areas of deglaciated orogens and ice sheets is mainly determined by geological and topographic conditions. Relatively broad ridges with steepened slopes and sufficient local relief formed by volcanic and sedimentary rocks are the most likely regions for large landslides in Patagonia. Long-term glacial erosion leading to the exposure of strong crystalline basement and the formation of U-shaped valleys separated by narrow ridges reduces the chance of the genesis of large (km-scale) landslides. We explain the different distribution of landslides in the study area compared to other mountain ranges where large landslides occupy mostly the highest and steepest ridges^57^ by the coincidence of four specific features of the Patagonian and Fuegian Andes: (1) the exceptionally long glaciation, (2) the presence of one of the largest and thickest Quaternary ice sheets in the world, (3) the existence of one of the world's largest resilient granite massifs, which builds most of the alpine part of the PIS, and 4) the development of large glacial lakes that, after the LGM, flooded much of the eastern margin of the PIS formed by weak volcanic and sedimentary rocks. Further research should test the extent to which the length of glaciation and the degree of development of glacial topography correlate with the density of large landslides on a sample of mountain ranges from different climatic and geological settings.

## Supplementary Information

Supplementary Figures.

Supplementary Table 1.

Supplementary Information 3.
