# Supplementary material for: Large landslides cluster at the margin of a deglaciated mountain belt
Source: Sci Rep. 2022 Apr 5;12:5658. doi: 10.1038/s41598-022-09357-9 (PMC8983719; doi:10.1038/s41598-022-09357-9)
Supplement: Supplementary file 1 — Supplementary Figures. [file 41598_2022_9357_MOESM1_ESM.docx]

**Supplementary figures**

**
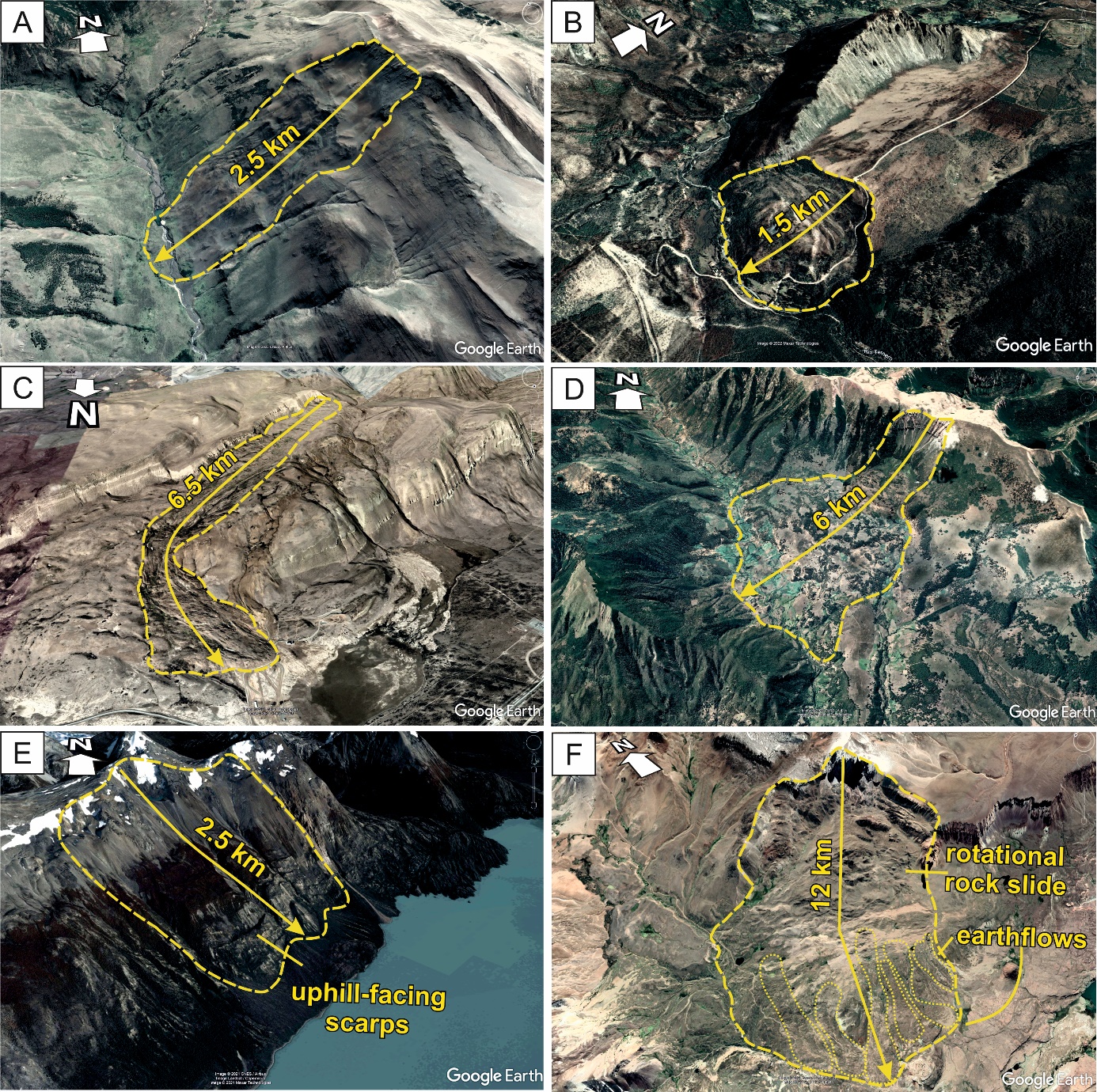
**

**Supplementary Fig. 1.** Google Earth^TM^ images of landslide types recognized in the PIS region. A. Rock slide in sedimentary rocks in the vicinity of Lago Argentino lake (Estancia Cristina site; Argentina); note partial damming of valley floor (ID_1506). B. Deep-seated debris slide affecting glacial deposits ~10 km to the east from El Bolsón, Argentina (ID_90). C. Earthflow descending from the basaltic mesa flanking the southern shore of the Lago Argentino lake; ~6 km to the east from El Calafate, Argentina (ID_640). D. Rock avalanche affecting effusive volcanic rocks in the Ránquil valley, Chile (ID_4). E. Deep-seated gravitational slope deformation in metamorphic rocks flanking slope of deep embayment of the Lago O'Higgins; ~8 km south from O'Higgins town, Chile (ID_460). F. Landslide complex involving rotational rock slide in basaltic rocks and distal earthflows partly affecting underlying Miocene sedimentary rocks along the western rim of the Meseta del Lago Buenos Aires, Argentina (ID_1683).

**
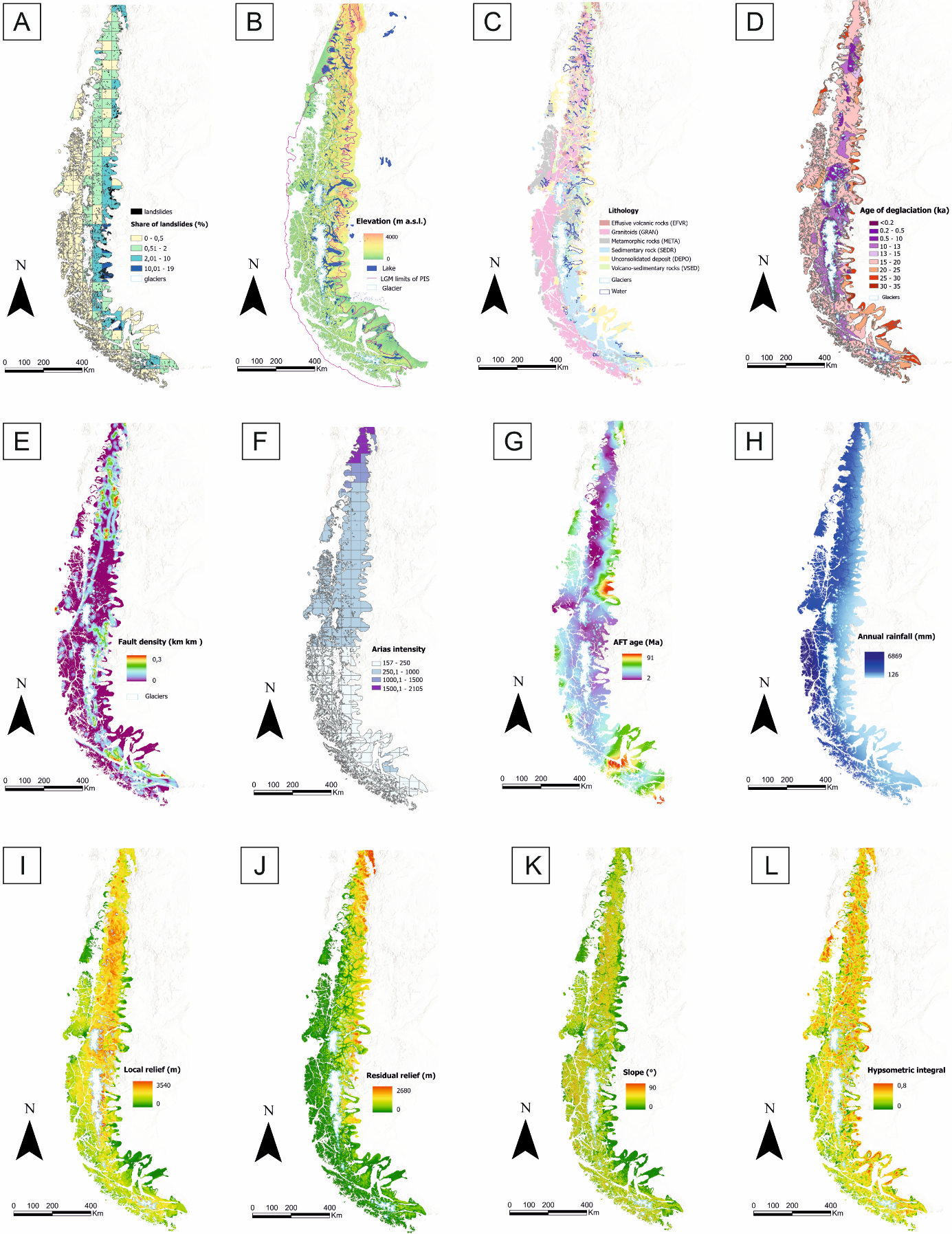
**

**Supplementary Fig. 2.** Selected landscape characteristics of PIS region. A. Landslide coverage (%) in 50-km square grid. B. Hypsometry calculated from 30-m NASADEM. C. Lithology classified into six main groups of rocks. D. Age of deglaciation. E. Fault density. F. Arias Intensity. G. Apatite Fission Track age. H. Mean annual precipitation. I. Local relief calculated within 5-km radius. J. Residual relief calculated by subtracting the base level from the topography. K. Slope calculated from 30-m NASADEM. L. Hypsometric integral calculated within 5-km radius.

**
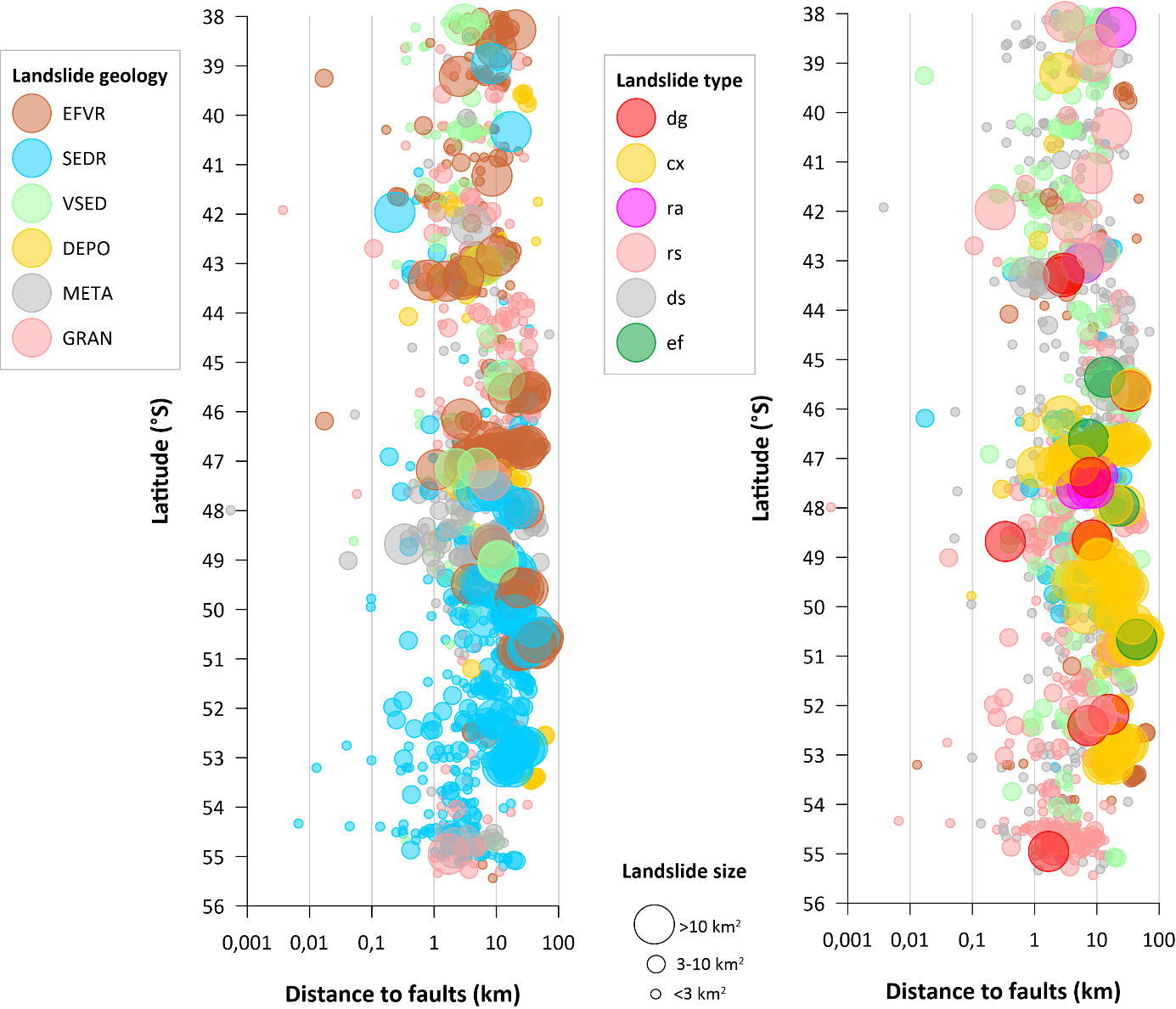
**

**Supplementary Fig. 3.** Distance of landslides from faults plotted as a function of latitude and landslide size. The left plot shows the lithology of the landslide, the right plot shows the type of landslide. Only a few large landslides overlap with mapped faults.

**
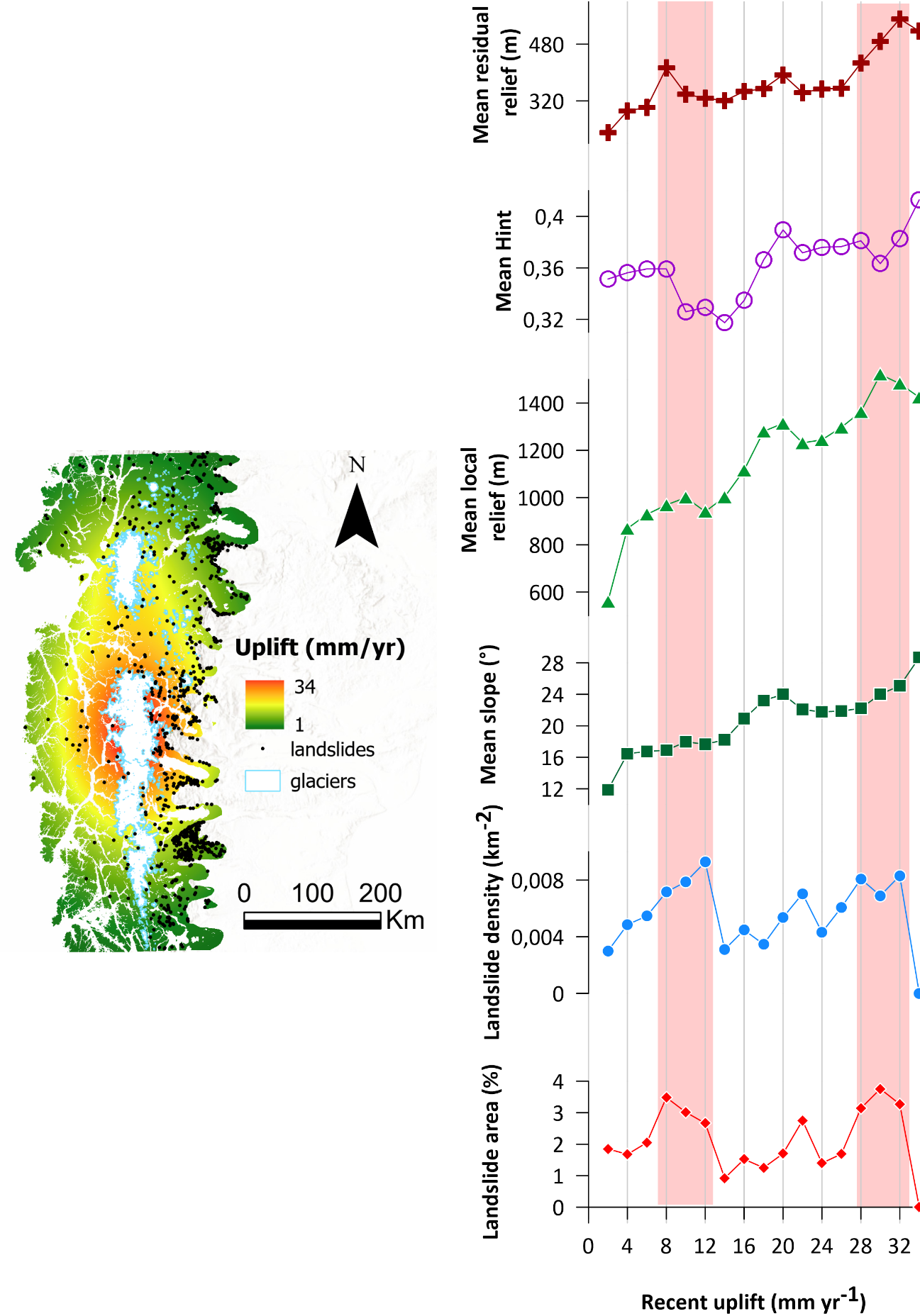
**

**Supplementary Fig. 4.** Comparison of recent uplift rates^23^ with landslide area/density and selected morphometric characteristics in the central Patagonian Andes (North and South Patagonian Icefields region). The occurrence of large landslides does not correlate with recent glacial isostatics uplift.

**
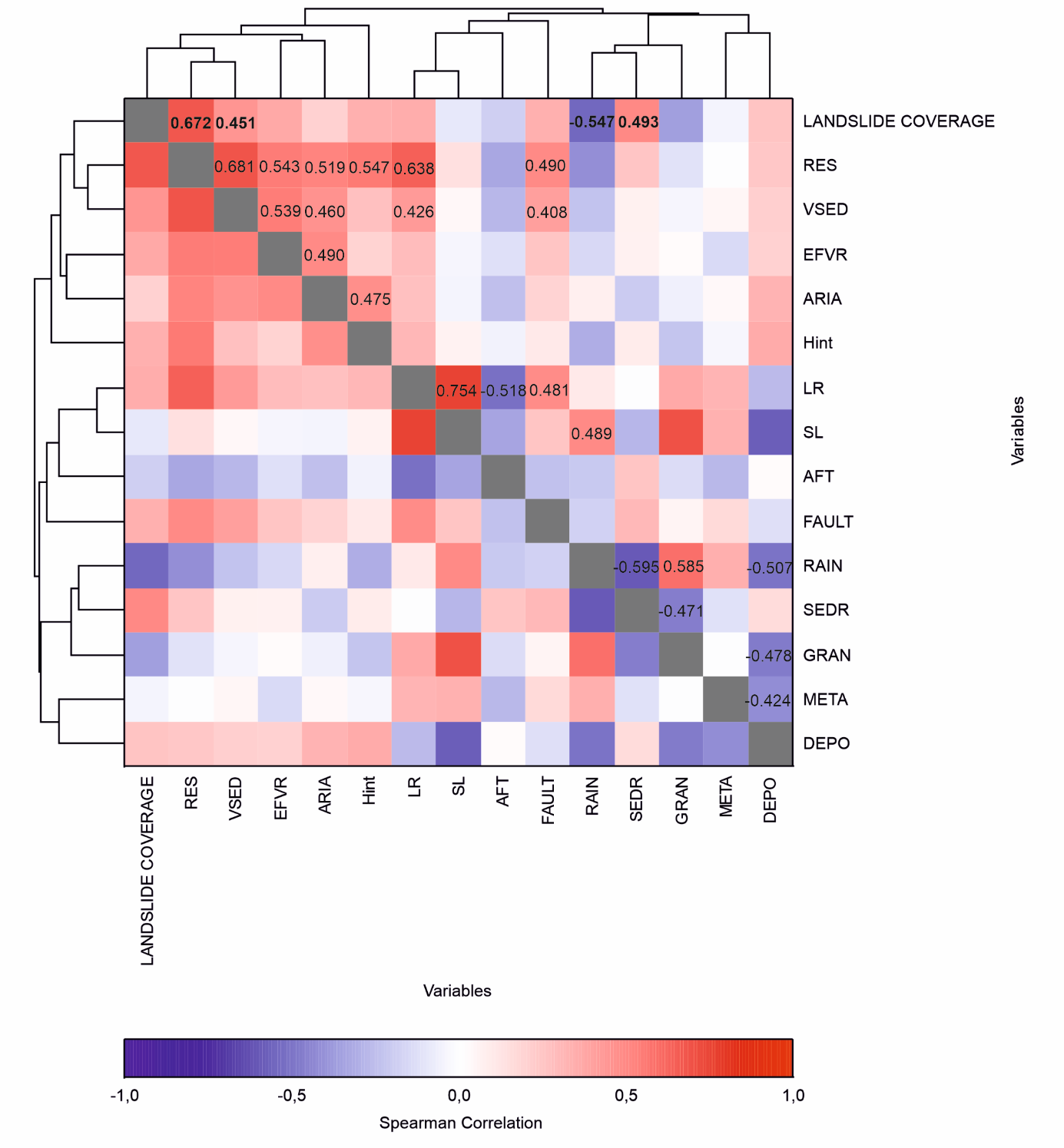
**

**Supplementary Fig. 5.** Heat map of the Spearman Correlation Matrix between variables calculated in 50-km square grids. Spearman's rank correlation coefficient *r_s_*>0.4 are indicated (the values are in bold in the case of landslide area correlation).

**Supplementary tables**

**Supplementary Table 1.** Area and number of large landslides in the main lithological units of PIS.

**Supplementary data**

**Supplementary data 1.** Main characteristics of large landslides (>0.9 km^2^) mapped within the PIS region.
