# Supplementary material for: Large landslides cluster at the margin of a deglaciated mountain belt
Source: Sci Rep. 2022 Apr 5;12:5658. doi: 10.1038/s41598-022-09357-9 (PMC8983719; doi:10.1038/s41598-022-09357-9)
Supplement: Supplementary file 2 — Supplementary Table 1. [file 41598_2022_9357_MOESM2_ESM.docx]

|  | **Abbrev.** | **Area (km^2^)** | **% of mountain**  **area** | **Landslide area**  **(km^2^)** | **% of total**  **mountain area** | **Number of**  **landslides** | **% of landslide population** |
| --- | --- | --- | --- | --- | --- | --- | --- |
| **Total LGM Area** |  | 305178,2581 | 100 | 5928,07372 | 1,942495431 | 1457 | 100 |
| **Effusive volcanic rocks** | EFVR | 23039,97479 | 7,549677665 | 1923,917015 | 8,350343403 | 284 | 19,49210707 |
| **Granitoids** | GRAN | 121320,7142 | 39,75404899 | 502,4396943 | 0,414141721 | 204 | 14,00137268 |
| **Metamorphic rocks** | META | 44925,84568 | 14,72118163 | 559,2814246 | 1,244899047 | 180 | 12,35415237 |
| **Sedimentary rocks** | SEDR | 53785,08997 | 17,62415524 | 2244,994263 | 4,174008566 | 596 | 40,90597117 |
| **Unconsolidated deposits** | DEPO | 46407,05655 | 15,20654087 | 231,7532955 | 0,499392361 | 59 | 4,049416609 |
| **Volcano-sedimentary rocks** | VSED | 15699,57691 | 5,14439561 | 465,6880283 | 2,96624572 | 134 | 9,196980096 |
